# DPIs cannot replace pMDIs in the management of obstructive airways disease

**DOI:** 10.1007/s43678-025-00968-5

**Published:** 2025-07-22

**Authors:** Alan Kaplan

**Affiliations:** 1 Family Physician Airways Group of Canada, Aurora, ON Canada; 2 Family and Community Medicine, University of Toronto, Toronto, ON Canada

**Keywords:** Climate, Asthma, COPD, Inhaler, Climat, Asthme, BPCO, inhalateur

Thank you to Dr. Worrall for the article [1] ‘Just say no to metered dose inhalers’. We must attempt to improve planetary health, including by the reduction of greenhouse gases. However, the ‘one size fits all’ approach in advocating to replace all pressurized meter dose inhalers will harm patients who cannot use dry powder inhalers. Dry powder inhalers require a forceful inhalation to deaggregate larger powder particles to reach the lung, thus as stated by the authors, they are unsuitable for patients who cannot generate sufficient inspiratory flow (frail, elderly, very young, etc.) and also in those who have no alternative devices available such as those who are intubated. However, the authors’ overall conclusion that they are unable to find any ‘compelling reason’ to use metered dose inhalers does not sufficiently emphasize the individuality of these issues along with those of patient choice and comfort to optimize care.

In addition, some perspective is necessary, as pressurized MDIs currently account for only < 0.1% of the total greenhouse gases worldwide [2]. As mentioned by the authors, a dry powder inhaler has a carbon footprint of about 2% that of current pressurized meter dose inhalers; however, new propellants for these devices which have a reduced carbon footprint which are similar to the levels in dry powder inhalers are in development, with the first having recently receiving UK regulatory approval [3].

The authors appropriately suggest ensuring proper diagnosis, improving disease control/management to reduce use of rescue inhalers which is the biggest driver of inappropriate inhaler use, as well as proper recycling for optimal disposition. Optimal device use will ensure optimal drug delivery, improve efficacy, and reduce rescue medication use and importantly also exacerbations (a huge producer of greenhouse gases). Using spacer devices with the pressurized meter dose inhalers will further optimize delivery and also reduce emissions, with patients getting relief sooner and thus requiring fewer puffs of reliever.

The Global Initiative for Asthma (GINA) states, “Clinicians need to be aware of the potential to place an additional burden on patients of so called “green guilt”, which could impact negatively on adherence and increase the risk of exacerbations.” Selecting the right inhaler for each individual patient along with adequate education [4] is crucial to reduce patients’ symptom burden, and the need for emergency healthcare and hospitalization, all of which causes significantly greater environmental impacts than with the use of current pressurized meter dose inhalers. Forced switching of patients has been shown in multiple studies to lead to poorer control and exacerbations [5]. I will conclude with ‘The best and greenest inhaler is the one a patient can and will use’.
